# Supplementary material for: Historical trends in health care-related financial holdings among members of Congress
Source: PLoS One. 2021 Jul 21;16(7):e0253624. doi: 10.1371/journal.pone.0253624 (PMC8294517; doi:10.1371/journal.pone.0253624)
Supplement: S1 File — (DOCX) [file pone.0253624.s001.docx]

**APPENDIX A1.**

**DATA AND METHODOLOGY**

We obtained 2004-2014 data on the financial assets held by members of Congress from data files made available by the Center for Responsive Politics (CRP), a nonpartisan research group that tracks the flow of money in politics.^1,2^ CRP digitizes information from the required annual reports filed by members of Congress on their personal asset holdings.

We identified health care assets by initially identifying all assets classified by CRP as being in the health-related subsectors of the health, education, and human resources sector; the insurance subsector of finance, insurance, and real estate sector, and the drug store subsector of the general commerce sector. The full list of CRP sectors and their codes are listed below.

| **CRP Code** | **Description** |
| --- | --- |
| G4900 | Drug stores |
| F3200 | Accident & health insurance |
| H0000 | Health, Education & Human Resources |
| H1000 | Health professionals |
| H1100 | Physicians |
| H1110 | Psychiatrists & psychologists |
| H1120 | Optometrists & ophthalmologists |
| H1130 | Other physician specialists |
| H1400 | Dentists |
| H1500 | Chiropractors |
| H1700 | Other non-physician health practitioners |
| H1710 | Nurses |
| H1750 | Pharmacists |
| H2000 | Health care institutions |
| H2100 | Hospitals |
| H2200 | Nursing homes |
| H2300 | Drug & alcohol treatment hospitals |
| H3000 | Health care services |
| H3100 | Home care services |
| H3200 | Outpatient health services (incl drug & alcohol) |
| H3300 | Optical services (glasses & contact lenses) |
| H3400 | Medical laboratories |
| H3500 | AIDS treatment & testing |
| H3700 | HMOs |
| H3800 | Mental Health Services |
| H3900 | Health care Consultants |
| H4000 | Health care products |
| H4100 | Medical Devices & Supplies |
| H4200 | Personal health care products |
| H4300 | Pharmaceutical manufacturing |
| H4400 | Pharmaceutical wholesale |
| H4500 | Biotech products & research |

Because CRP sector identification was not comprehensive and firm names were not standardized (for example, CVS Caremark could be listed as "CVS", "CVS/Caremark", or "CVS Caremark") and included typographical errors, we standardized firm names in this initial list and identified additional health sector firms by matching these standardized names to firm names in the rest of the CRP data (i.e., those assets that may have received an "unknown" classification or a non-health sector classification).

To this augmented list, we added:

- any asset whose name included the string "HEALTH" or "MEDIC" and that, upon manual review, appeared to be a health care-related asset;
- any asset whose name matched the names of the top 5 electronic health record firms reimbursed by Medicare (these included Allscripts, Cerner, and athenahealth);
- any real estate asset identified as a medical practice, provider of health care services, skilled nursing facility, or a long-term care facility;
- investments in funds that specialized in health care, the life sciences, or biotechnology.

Because of inconsistencies in CRP sector classifications of assets, we re-classified assets using our own sector classification system, based on the North American Industry Classification System (NAICS). NAICS is a standard used by the US government and others to classify firms according to their business activity. Public firms on our asset list were assigned the NAICS classification associated with their firm listing on Compustat, a comprehensive database of publicly traded firms, with the exception of two firms whose sector of business activity had changed (e.g., from HMO to insurance) since they were first publicly listed. For privately traded firms, a NAICS classification was assigned by 3 research assistants, independently, based on information obtained through web and business listing searches. Conflicts in the classification of privately traded firms were adjudicated by two of the investigators (GPK and MM). We then aggregated the NAICS classifications into 9 broad sector classifications:

- health care, biotech, and life sciences funds;
- pharmaceuticals, biologics, and chemical and molecular diagnostics;
- medical devices, instruments, and supplies;
- health insurance carriers and pharmacy benefit managers;
- pharmacies and wholesale drug distributors;
- health care services, including hospitals, ambulatory care facilities, dialysis facilities, laboratories, and physician practices;
- administrative services, including information technology services, electronic health record systems, and health care consulting;
- real estate, including skilled nursing facilities and long-term care facilities
- miscellaneous, including contract research organizations.

Firms or funds whose identity could not be confirmed through Compustat or web searches, or that could not be unambiguously identified as health-related firms (for example, firms with common names) were excluded from the final list of health sector assets. We also excluded any health sector assets that were part of pensions or retirement accounts.

For calculations involving the asset values, we used the exact values of the assets when they were reported. When a range was reported instead, we used the mean of the lower and upper bounds of the reported range as indicated below:

| Asset Range | Value Used |
| --- | --- |
| >$0 - $1,000 | $500 |
| $1,001 - $15,000 | $8,000 |
| $15,001 - $50,000 | $32,500 |
| $50,001 - $100,000 | $75,000 |
| $100,001 - $250,000 | $175,000 |
| $250,001 - $500,000 | $375,000 |
| $500,001 - $1,000,000 | $750,000 |
| >$1,000,000 | $1,500,000 |

For the top asset category, for which there was no upper bound, we observed that there was a doubling in the substituted values for the 3 preceding categories—e.g., a $200,000 difference from $175,000 to $375,000, followed by a $400,000 difference from $375,000 to $750,000. Following this pattern, we assumed a doubling of the difference, to $800,000, so the value used for the >$1,000,000 asset range was $1,500,000. In asset value calculations, we excluded any income associated with the assets, as well as any income or losses associated with the sales of assets in any given year.

To maintain comparability in dollar values across years, we deflated all dollar amounts by the US Consumer Price Index: Total All Items (Series CPALTT01USA661S, obtained from Federal Reserve Economic Data).^3^ All dollar amounts were reported in 2019 US dollars.

**REFERENCES FOR APPENDIX**

1. Personal Finances. Center for Responsive Politics. [(8)](https://www.opensecrets.org/personal-finances).
2. Bulk Data. Center for Responsive Politics. <https://www.opensecrets.org/bulk-data>
3. FRED Economic Data. Federal Reserve Bank of St. Louis Economic Research. <https://fred.stlouisfed.org/>.
